# Multi-Matrices Factorization with Application to Missing Sensor Data Imputation

**DOI:** 10.3390/s131115172

**Published:** 2013-11-06

**Authors:** Xiao-Yu Huang, Wubin Li, Kang Chen, Xian-Hong Xiang, Rong Pan, Lei Li, Wen-Xue Cai

**Affiliations:** 1 Software Institute, Sun Yat-Sen University, Guangzhou 510275, China; E-Mails: panr@mail.sysu.edu.cn (R.P.); lnslilei@mail.sysu.edu.cn (L.L.); 2 School of Economics and Commerce, South China University of Technology, Guangzhou 510006, China; E-Mail: ctwxcai@scut.edu.cn; 3 Department of Computing Science, Umeå University, SE-901 87 Umeå, Sweden; E-Mail: wubin.li@cs.umu.se; 4 Academy of Guangdong Telecom Co.Ltd, Guangzhou 510630, China; E-Mail: chenkang@gsta.com; 5 Department of Interventional Radiology, the First Affiliated Hospital of Sun Yat-Sen University, Guangzhou 510080, China; E-Mail: med.interventional@163.com

**Keywords:** matrix factorization, sensor data, probabilistic graphical model, missing estimation

## Abstract

We formulate a multi-matrices factorization model (MMF) for the missing sensor data estimation problem. The estimation problem is adequately transformed into a matrix completion one. With MMF, an *n*-by-*t* real matrix, *R*, is adopted to represent the data collected by mobile sensors from *n* areas at the time, *T*_1_, *T*_2_, … , *T*_t_, where the entry, *R_i,j_*, is the aggregate value of the data collected in the *ith* area at *T_j_*. We propose to approximate *R* by seeking a family of *d*-by-*n* probabilistic spatial feature matrices, *U*_(1)_, *U*_(2)_, … , *U*_(_*_t_*_)_, and a probabilistic *temporal feature matrix*, *V* ∈ ℝ*^d^*^×^*^t^*, where $R_{j}\approx U_{\left(j\right)}^{T}T_{j}$. We also present a solution algorithm to the proposed model. We evaluate MMF with synthetic data and a real-world sensor dataset extensively. Experimental results demonstrate that our approach outperforms the state-of-the-art comparison algorithms.

## 1. Introduction

In this work, we study the following missing sensor data imputation problem: Let the matrix, *R* ∈ ℝ*^n^*^×^*^t^*, consist of the data collected by a set of mobile sensors in spacial areas *S*_1_, *S*_2_, … *S_n_* at time points *T*_1_ < *T*_2_ < … < *T_t_*, where the entry, *R_i,j_*, is the aggregate value collected by the sensors in *S_i_* at *T_j_*. In particular, if there is no sensor in *S_i_* at time *T_j_*, we denote the value of *R_i,j_* as “ ⊥ ”, which indicates that it is *missing*. Our focus is to find the suitable estimations for the missing values in a given incomplete matrix, *R*. Results of this research could be helpful in recovering missing values in statistical analyses. For example, to predict floods, people usually place geographically distributed sensors in the water to continuously monitor the rising water levels. However, some data in a critical period of time might be corrupted, due to, e.g., sensor hardware failures. Such a kind of data needs be recovered to guarantee the prediction accuracy.

Many efforts have been devoted to the missing sensor data imputation problem. Typical examples include *k* nearest neighbor-based imputation [1], multiple imputation [2], hot/cold imputation [3], maximum likelihood and Bayesian estimation [4] and expectation maximization [5]. However, despite the various implementations of these methods, their main essence is based on the *local consistency* of the sensor data, *i.e.*, the data collected at adjacent time points within the same spacial area should be close to each other, as well as the data collected at the same time from neighboring areas. We refer to them as *local models*. As is well known, these local models suffer from the cumulative error problem in scenarios where the missing ratio is high.

Matrix factorization (MF), as a *global model*, has caught substantial attention in recent years. Typically, in the *Netflix* rating matrix completion competition [6], some variations of the MF model, e.g., [7,8], achieved state-of-the-art performances, showing their potential to recover the missing data from highly incomplete matrices. On the other side, many well-studied MF models, such as non-negative matrix factorization [9], max margin matrix factorization [10,11], and probabilistic matrix factorization [7], are based on the i.i.d.assumption [12], which, in terms of our problem, implies that the neighborhood information among the data is disregarded and, hence, leaves vast room for improvement.

We in this paper, we propose a multi-matrices factorization model (MMF), which can be outlined as follows. For a matrix, *X*, denote *X_j_* the *jth* column of *X*. Given a sensor data matrix, *R*, we seek a set of matrices, *U*_(1)_, *U*_(2)_, …, *U*_(*t*)_ ∈ ℝ*^d^*^×^*^n^*, and a matrix, *V* ∈ ℝ *^d^*^×^*^t^*, such that for *i* = 1, 2, …,*t*, $U_{\left(i\right)}^{T}V_{i}\approx R_{i}$. Here, *U*_(_*_i_*_)_ is referred to as the *spatial feature matrix*, in which the *jth* column, *U*_(_*_i_*_)_*_,j_*, is the feature vector of area *S_j_* at *T_i_*. Similarly, *V* is referred to as the *temporal feature matrix*, in which the *jth* column, *V_j_*, is the temporal feature vector of *T_j_*. To predict the missing values in *R*, we first fit the matrices, *U*_(1)_, *U*_(2)_, …, *U*_(_*_t_*_)_ (single sub-indexes of matrix mean columns) and *V*, with the non-missing values in *R*; then, for each *R_i,j_* = “ ⊥ ”, we take its estimation as ${\hat{R}}_{i,j}=U_{(j),i}^{T}V_{j}$.

The remainder of the paper is organized as follows: Section 2 summarizes the notations used in the paper. Section 3 studies the related work on matrix factorization. In Section 4, we present our multi-matrices factorization model. The algorithm to solve the proposed model is outlined in Section 5. Section 6 is devoted to the experimental evaluations. Finally, our conclusions are presented in Section 7, followed by a presentation of future work, acknowledgments and a list of references.

## 2. Notations

For a vector *V* = [*v*_1_*, v*_2_*, …v_n_*]*′* ∈ ℝ*^n^*, we use ‖*V*‖_0_, ‖*V*‖_1_ and ‖*V*‖_2_ to denote its 0-Norm, 1-Norm and 2-Norm, respectively, as follows:

- ${\left\|V\right\|}_{0}=\sum_{i=1}^{n}\text{I}({\text{v}}_{i}\neq 0)$, *i.e.*, the number of nonzero entries of *V*;
- ${\left\|V\right\|}_{1}=\sum_{i=1}^{n}\left|v_{i}\right|$;
- ${\left\|V\right\|}_{2}=\sqrt{\sum_{i=1}^{n}v_{i}^{2}}$.

For a matrix, *X* ∈ ℝ*^n^*^×^*^m^*, we denote its *Frobenius* norm as ${\left\|X\right\|}_{F}=\sqrt{\sum_{i=1}^{n}\sum_{j=1}^{m}X_{i,j}^{2}}$.

## 3. Matrix Factorization

The essence of the *Matrix Factorization* problem is to find two factor matrices, *U* and *V*, such that their product can approximate the given matrix, *R*, *i.e.*, *R* ≈ *U^T^V*. As a fundamental model of machine learning and data mining, the MF method has achieved state-of-the-art performance in various applications, such as collaborative filtering [13], text analysis [14], image analysis [9,15] and biology analysis [16]. In principle, for a given matrix, *R*, the MF problem can be formulated as the optimization model below:

(1) $$\left\{U^{*},V^{*}\right\}=\underset{U,V}{\text{min}}\text{Loss}(U^{T}V,R)$$

where the lose function, Loss, is used to measure the closeness of the approximation, *U^T^V*, to the target, *R*. Usually, *Loss*(*U^T^V*, *R*) can be decomposed into the sum of the pairwise loss between the entries of *U^T^V* and *R*; that is, $\text{Loss}\left(U^{T}V,R\right)=\sum_{i=1}^{n}\sum_{j=1}^{m}\text{loss}\left({\left(U^{T}V\right)}_{i,j},R_{i,j}\right)$. Some of the most used forms include the square loss (*loss*(*x*, *y*) = (*x* - *y*)^2^) [7,8,17], the 0-1 loss (*loss*(*x*, *y*) = 𝕀(*x* = *y*)) [11] and the divergence loss $\left(\text{loss}\left(x,y\right)=\text{xlog}\frac{x}{y}-x+y\right)$ [9].

It is notable that for Equation (1), if {U*, V*} is a solution to it, then for any scalar, *κ* > 0, $\left\{\kappa U^{*},\frac{1}{\kappa }V^{*}\right\}$ is also another solution; hence, problem (1) is ill-posed. To overcome this obstacle, various constraints on *U* and *V* are introduced, such as constraints on the entries [15], constraints on the sparseness [18,19], constraints on the norms [7,20] and constraints on the ranks [21,22]. All these constraints, from the perspective of the statistical learning theory, can be regarded as the length of the model to be fitted. According to the minimum description length principle [23,24], a smaller length means a better model; hence, most of them can be incorporated into Model (1) as the additional regularized terms, that is:

(2) $$\left\{U^{*},V^{*}\right\}=\underset{U,V}{\text{min}}\text{Loss}\left(U^{T}V,R\right)+P\left(U,V\right)$$

where the regularization factor, *P*(*U*, *V*), corresponds to the constraints on *U* and *V* .

As a transductive model, Model (2) has many nice mathematical properties, such as the generalization error bound [10] and the exactness [17,25]. However, as is well known, when compared with the generative model, one of the main restrictions of the transductive model is that it can hardly be used to describe the relations existing in the data. In particular, in terms of our problem, even though Model (2) may work well, it is laborious to express the dynamics of the data over time.

## 4. The Proposed Model

In this section, we elaborate on our multi-matrices factorization (MMF) approach. Given the sensor data matrix, *R*, in which the entry, *R_i,j_* (1 ≤ *i* ≤ *n* and 1 ≤ *j* ≤ *t*), is collected from *S_i_* at *T_j_*, our goal is to find the factor matrices, *U*_(1)_, *U*_(2)_, …, *U*_(_*_t_*_)_ ∈ ℝ*^d^*^×^*^n^* and *V* ∈ ℝ*^d^*^×^*^t^*, such that: for *j* = 1, 2, …, *t*,

(3) $$R_{j}\approx U_{\left(j\right)}^{T}V_{j}$$

where *U*_(_*_j_*_)_ is regarded to be composed of the spatial features of all areas at *T_j_* and *V* is treated as consisting of the temporal features of all time points. We denote the *i* th column of *U_j_* as *U*_(_*_j_*_)_,_*i*_, which corresponds to the spatial feature value of *S_i_* at *T_j_*, and denote the *j* th column of *V* as *V_j_*, which corresponds to the temporal feature value of *T_j_*.

**Figure 1.**
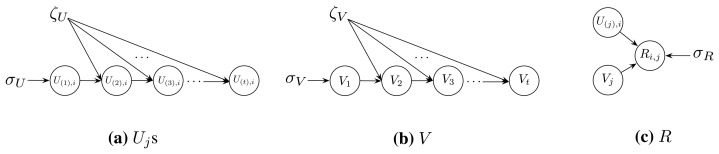
The structure assumptions.

Taking advantage of the knowledge of the probability graph model, we assume that the dependent structure of the data in *U*_(1)_, *U*_(2)_, …, *U*_(_*_t_*_)_, *V* and *R* is as illustrated in Figure 1. More specifically, we have the following assumptions:

- Columns of *U*_(_*_j_*_)_ (1 ≤ *j* ≤ *t*) are linearly independent, *i.e.*,
  $$Pr\left(U_{\left(j\right)}\right)=\prod_{i=1}^{n}Pr\left(U_{(j),i}\right)$$
- *U*_(1),_*_i_* (1 ≤ *i* ≤ *n*) follows the same Gaussian distribution with a mean of zero and a covariance matrix $\sigma_{U}^{2}I$, *i.e.*,
  $$\text{Pr}\left(U_{(1),i}|\sigma_{U}\right)={\left(2\pi \sigma_{U}^{2}\right)}^{-\frac{d}{2}}\text{exp}\{-\frac{{\left\|U_{(1),i}\right\|}_{2}^{2}}{2\sigma_{U}^{2}}\}$$
- *U*_(_*_j_*_),__*i*_ (1 ≤ *i* ≤ *n*) are dependent in time order with the pre-specified priors, *ζ_U_* and *σ_U_*, *i.e.*,
  $$\text{Pr}\left(U_{(j),i}|\zeta_{U},\sigma_{U}\right)=\text{Pr}\left(U_{(1),i}|\zeta_{U},\sigma_{U}\right)\times \prod_{j=2}^{t}\text{Pr}\left(U_{(j),i}|U_{(j-1),i},\zeta_{U},\sigma_{U}\right)$$
  Moreover, for *j* > 1, we assume *U*_(*j*),_*_i_* is a Laplace random vector with location parameter *U*_(*j*-1),_*_i_* and scale parameter *ζ_U_*, namely:
  $$\text{Pr}\left(U_{(j),i}|U_{(j-1),i},\zeta_{U},\sigma_{U}\right)=\frac{1}{2\zeta_{U}}\text{exp}\{-\frac{\left|U_{(j),i}-U_{(j-1),i}\right|}{\zeta_{U}}\}$$
- The columns of *V* are linearly dependent in time order with the pre-specified priors, *ζ_V_* and *σ_V_*, *i.e.*,
  $$\text{Pr}\left(V|\zeta_{V},\sigma_{V}\right)=\text{Pr}\left(V_{1}|\zeta_{V},\sigma_{V}\right)\times \prod_{j=2}^{t}\text{Pr}\left(V_{j}|V_{j-1},\zeta_{V},\sigma_{V}\right)$$
  We also assume that, for *j* > 1:
  $$\text{Pr}\left(V_{j}|V_{j-1},\zeta_{V},\sigma_{V}\right)=\frac{1}{2\zeta_{V}}\text{exp}\{-\frac{\left|V_{j}-V_{j-1}\right|}{\zeta_{V}}\}$$
- The (*i*, *j*)*th* entry of *R* (1 ≤ *i* ≤ *n*, 1 ≤ *j* ≤ *t*) follows Gaussian distribution with a mean of $U_{(j),i}^{T}V_{j}$ and variance $\sigma_{R}^{2}$, *i.e.*,
  $$\text{Pr}\left(R_{i,j}|U_{(j),i}^{T}V_{j},\sigma_{R}^{2}\right)={\left(2\pi \sigma_{R}^{2}\right)}^{-\frac{1}{2}}\text{exp}\{-\frac{{\left(R_{i,j}-U_{(j),i}^{T}V_{i}\right)}^{2}}{2\sigma_{R}^{2}}\}$$

Now, given *R* and the priors, *σ_U_*, *σ_V_*, *σ_R_*, *ζ_U_*, and *ζ_V_*, let *U* = {*U*_(1)_, *U*_(2)_, …, *U*_(_*_t_*_)_}; below, we find a solution to the following equation:

(4) $$\{U^{*},V^{*}\}=\underset{U,V}{\text{arg}}\text{maxPr}\left(U,V|R,\sigma_{U},\sigma_{V},\sigma_{R},\zeta_{U},\zeta_{V}\right)$$

First, applying Bayes' theorem, we have:

$$\text{Pr}\left(U,V|R,\sigma_{U},\sigma_{V},\sigma_{R},\zeta_{U},\zeta_{V}\right)=\frac{\text{Pr}\left(U,V,R|\sigma_{U},\sigma_{V},\sigma_{R},\zeta_{U},\zeta_{V}\right)}{\text{Pr}\left(R|\sigma_{U},\sigma_{V},\sigma_{R},\zeta_{U},\zeta_{V}\right)}$$

Since *R* is observed and *σ_U_*, *σ_V_*, *σ_R_*, *ζ_U_* and *ζ_V_* are pre-specified, the denominator, *Pr*(*R*∣*σ_U_*, *σ_V_*, *σ_R_*, *ζ_U_*, *ζ_V_*), can be treated as a constant. Therefore:

(5) $$\begin{array}{l} \text{Equation}(4)\Leftrightarrow \\ \{U^{*},V^{*}\}=\underset{U,V}{\arg}\text{maxPr}\left(U,V,R|\sigma_{U},\sigma_{V},\sigma_{R},\zeta_{U},\zeta_{V}\right) \end{array}$$

Combing Assumptions (I.) ∼ (V.) and the dependency structure illustrated in Figure 1, we have:

$$\begin{array}{l} \text{Pr}\left(R,U,\text{V}|\sigma_{U},\sigma_{V},\sigma_{R},\zeta_{U},\zeta_{V}\right)\\ =\text{Pr}\left(R|U,V,\sigma_{U},\sigma_{V},\sigma_{R},\zeta_{U},\zeta_{V}\right)\times \text{Pr}\left(U,V|\sigma_{U},\sigma_{V},\sigma_{R},\zeta_{U},\zeta_{V}\right)\\ =\text{Pr}\left(R|U,V,\sigma_{R}\right)\times \text{Pr}\left(U|\sigma_{U},\zeta_{U}\right)\times \text{Pr}\left(V|\sigma_{V},\zeta_{V}\right)\\ =\overset{n}{\underset{i=1}{\text{∏}}}\overset{t}{\underset{j=1}{\text{∏}}}\text{Pr}\left(R_{i,j}|U_{(j),i},V_{j},\sigma_{R}\right)\times \overset{t}{\underset{j=1}{\text{∏}}}\text{Pr}\left(U_{\left(j\right)}|\sigma_{U},\zeta_{U}\right)\times \text{Pr}\left(V|\sigma_{V},\zeta_{V}\right)\\ =\overset{n}{\underset{i=1}{\text{∏}}}\overset{t}{\underset{j=1}{\text{∏}}}\text{Pr}\left(R_{i,j}|U_{(j),i},V_{j},\sigma_{R}\right)\times \overset{t}{\underset{j=1}{\text{∏}}}\text{Pr}\left(U_{(j),1}|\sigma_{U}\right)\overset{n}{\underset{i=1}{\text{∏}}}\overset{t}{\underset{j=2}{\text{∏}}}\text{Pr}\left(U_{(j),i}|U_{(j-1),i},\zeta_{U}\right)\times \text{Pr}(V_{1}|\sigma_{V})\times \overset{t}{\underset{j=2}{\text{∏}}}\text{Pr}\left(V_{j}|V_{j-1},\zeta_{V}\right)\\ \propto \exp(-\frac{1}{2\sigma_{R}^{2}}\overset{n}{\underset{i=1}{\text{∑}}}\overset{t}{\underset{j=1}{\text{∑}}}{\left(U_{(j),i}^{T}V_{i}-R_{i,j}\right)}^{2}))\times \exp(-\frac{1}{2\sigma_{U}^{2}}\overset{n}{\underset{i=1}{\text{∑}}}{\left\|U_{(i),1}\right\|}_{2}^{2})\times \overset{n}{\underset{i=1}{\text{∏}}}\exp(-\overset{t}{\underset{j=2}{\text{∑}}}\frac{\left|U_{(j),i}-U_{(j-1),i}\right|}{\zeta_{U}})\times \exp(-\frac{1}{2\sigma_{V}^{2}}{\left\|V_{1}\right\|}_{2}^{2})\times \exp(-\overset{t}{\underset{j=2}{\text{∑}}}\frac{\left|V_{j}-V_{j-1}\right|}{\zeta_{V}}) \end{array}$$

Taking the logarithm on both sides and take the missing values into account, then we have:

(6) $$\begin{array}{l} \text{Equation}(5)\Leftrightarrow \\ \{U^{*},V^{*}\}=\underset{U,V}{\text{arg}}\min\left\{\overset{n}{\underset{i=1}{\text{∑}}}\overset{t}{\underset{j=1}{\text{∑}}}{\left({\text{R}}_{i,j}-U_{(j),i}^{T}V_{j}\right)}^{2}\text{I}(R_{i,j}\neq^{\prime }{⊥}^{\prime })+\alpha \overset{t}{\underset{j=1}{\text{∑}}}{\left\|U_{(j),1}\right\|}_{2}^{2}+\gamma \overset{n}{\underset{i=1}{\text{∑}}}\overset{t}{\underset{j=2}{\text{∑}}}{\left\|U_{(j),i}-U_{(j-1),i}\right\|}_{1}+\beta {\left\|V_{1}\right\|}_{2}^{2}+\lambda \overset{t}{\underset{j=2}{\text{∑}}}{\left\|V_{j}-V_{j-1}\right\|}_{1}\right\} \end{array}$$

where $\alpha =\frac{\sigma_{R}^{2}}{\sigma_{U}^{2}}$, $\gamma =\frac{\sigma_{R}^{2}}{\zeta_{U}}$, $\beta =\frac{\sigma_{R}^{2}}{\sigma_{V}^{2}}$ and $\lambda =\frac{\sigma_{R}^{2}}{\zeta_{V}}$ are the regularization parameters.

As a supplement, we have the following comments on Model (6):

- *On the selection of the Gaussian prior:* In our model, since no prior information is available for the columns of the matrices, *U*_(1)_ and *V*, hence, according to the max entropy principle [26], a reasonable choice for them is the Gaussian prior distribution.
- *On the ability to formalize the dynamics of the sensor data*: The ability to characterize the dynamics of the sensor data lies in the terms $\gamma {\sum_{i=1}^{n}\sum_{j=2}^{t}\left\|U_{(j),i}-U_{(j-1),i}\right\|}_{1}$ and $\lambda {\sum_{j=2}^{t}\left\|V_{j}-V_{j-1}\right\|}_{1}$. Obviously, for any two adjacent time points, *T_j_*_−1_ and *T_j_*, if the interval is small enough (namely, |*T_j_* – *T_j_*_−1_| → 0), then for any area, *S_i_*, the values, *R_i,j_*_−1_ and *R_i,j_*, should be close to each other (namely, |*R_i,j_* – *R_i,j_*_−1_| → 0). This can been enforced by tuning the parameters, γ and λ (see the following elaboration).
  First of all, since for any *x* ∈ ℝ*^n^*, ‖*x*‖_2_ ≤ ‖*x*‖_1_, we have:
  (7) $$\begin{array}{ll} {\left\|R_{i,j}-R_{i,j-1}\right\|}_{2} & ={\left\|U_{(j),i}^{T}V_{j}-U_{(j-1),i}^{T}V_{j-1}\right\|}_{2}\\ & ={\left\|{\left(U_{(j),i}-U_{(j-1),i}\right)}^{T}V_{j}+U_{(j-1),i}^{T}(V_{j}-V_{j-1})\right\|}_{2}\\ & \leq {\left\|U_{(j),i}-U_{(j-1),i}\right\|}_{2}{\left\|V_{j}\right\|}_{2}+{\left\|U_{(j-1),i}\right\|}_{2}{\left\|V_{j}-V_{j-1}\right\|}_{2}\\ & \leq {\left\|U_{(j),i}-U_{(j-1),i}\right\|}_{1}{\left\|V_{j}\right\|}_{2}+{\left\|U_{(j-1),i}\right\|}_{2}{\left\|V_{j}-V_{j-1}\right\|}_{1} \end{array}$$
  Secondly, it is obvious that greater regularization parameters (*i.e.*, *α*, *β*, *γ* and λ) result in smaller corresponding multipliers (*i.e.*, ${\sum_{j=1}^{t}\left\|U_{(j),1}\right\|}_{2}^{2}$, ${\left\|V_{1}\right\|}_{2}^{2}$, $\sum_{i=1}^{n}\sum_{j=2}^{t}{\left\|U_{(j),i}-U_{(j-1),i}\right\|}_{1}$ and $\sum_{j=2}^{t}{\left\|V_{j}-V_{j-1}\right\|}_{1}$. In particular:
  (8) $$\gamma \to \infty \Rightarrow {\left\|U_{(j),i}-U_{(j-1),i}\right\|}_{1}\to 0$$
  and:
  (9) $$\lambda \to \infty \Rightarrow {\left\|V_{j}-V_{j-1}\right\|}_{1}\to 0$$
  Hence, combining Equations (7)-(9), when |*T_j_* – *T_j_*_−1_| → 0, we can simply take *γ* → ∞ and λ → ∞ and achieve ‖*R_j_* – *R_j_*_−1_‖_2_ → 0.
  On the other side, when |*T_j_* – *T_j_*_−1_| → ∞, as is well known, the values in *R_j_* and *R_j_*_−1_ are regarded as being independent. In this case, we can take *γ* → 0 and λ → 0, allowing *R_j_* to be irrelevant to *R*_*j*−1_.
- *On the* ℓ_1_ *norm:* It is straightforward to verify that, if we replace the ℓ_1_ terms in Equation (6) with the ℓ_2_ terms (equivalently, use the Gaussian distribution instead of the Laplace distribution in Assumptions (III.) and (IV.)), e.g., replacing ‖*V_j_* – *V_j_*_−1_‖_1_ with ${\left\|V_{j}-V_{j-1}\right\|}_{2}^{2}$, ‖*R_j_* – *R_j_*_−1_‖_2_ can still be bounded via tuning the regularization parameters, *γ* and λ. The reason for adopting the ℓ_1_ norm here is two-fold: Firstly, as shown above, the ℓ_1_ terms can lead to the bounded difference norm, ‖*R_j_* – *R_j_*_−1_‖_2_, and hence, the proposed model accommodates the ability to characterize the dynamics of the sensor data; secondly, according to the recent emerging works on compressed sensing [27,28], under some settings, the behavior of the ℓ_1_ norm is similar to that of the ℓ_0_ norm. In terms of our model, this result indicates that the ℓ_1_ terms can restrict not only the magnitudes of the dynamics happening to the features, but also the number of features that changed in adjacent time points. In other words, with ℓ_1_ norms, our model gains more expressibility.

## 5. The Algorithm

Below, we present the algorithm to solve Model (6). We denote:

$$W=\underset{U,V}{\text{arg}}\min\overset{n}{\underset{i=1}{\text{∑}}}\overset{t}{\underset{j=1}{\text{∑}}}{\left(R_{i,j}-U_{(J),i}^{T}V_{j}\right)}^{2}\text{I}(R_{i,j}\neq^{\prime }{⊥}^{\prime })+\alpha \overset{t}{\underset{j=1}{\text{∑}}}{\left\|U_{(j),1}\right\|}_{2}^{2}+\beta {\left\|V_{1}\right\|}_{2}^{2}+\gamma \overset{n}{\underset{i=1}{\text{∑}}}\overset{t}{\underset{j=2}{\text{∑}}}{\left\|U_{(j),i}-U_{(j-1),i}\right\|}_{1}+\lambda \overset{t}{\underset{j=2}{\text{∑}}}{\left\|V_{j}-V_{j-1}\right\|}_{1}$$

Apparently, *W* is convex with respect to *U*_(_*_j_*_),_*_i_* and *V_j_* (1 ≤ *i* ≤ *n* and 1 ≤ *j* ≤ *t*). Therefore, we can obtain the local minimum solution via coordinate descent [29].

First, we introduce the signum function, *sgn*, for a real variable, *x*:

$$\text{sgn}(x)=\{\begin{array}{ll} 1 & \text{if}\;x>0\\ -1 & \text{if}\;x<0\\ 0 & \text{if}\;x=0 \end{array}$$

For *X* = [*x*_1_, *x*_2_, …*x_n_*]′ ∈ ℝ*^n^*, we denote *sgn*(*X*) *=* [*sgn*(*x*_1_), *sgn*(*x*_2_), …, *sgn*(*x_n_*)]′.

Then, we calculate the partial subgradient of *W* with regard to *U*_(_*_j_*_)_*_,i_* (1 ≤ *i* ≤ *n* and 1 ≤ *j* ≤ *t*) as follows:

For *j* = 2, 3, …, *t*, define *F_j_*_,_*_i_*_,1_ = *γsgn*(*U*_(_*_j_*_),_*_i_* – *U*_(_*_j_*_−1),_*_i_*).
For *j* = 1, 2, …, *t* – 1, define *F_j_*_,_*_i_*_,2_ = –*γF_j_*_+1,_*_i_*_,1_.
Let *F*_1,_*_i_*_,1_ = *F_t,i,2_*= 0, and we have:
For *i* = 1, 2 …,*n*:
  $$\frac{\partial W}{\partial U_{(1),i}}=2\alpha U_{(1),i}-2(R_{i,1}-U_{(1),i}^{T}V_{j})V_{j}\text{I}(R_{i,j}\neq^{\prime }{⊥}^{\prime })+F_{1,i,1}+F_{1,i,2}$$
For *i* = 1,2 …,*n* and *j* = 2, 3, …, *t*:
  $$\frac{\partial W}{\partial U_{(j),i}}=-2({\text{R}}_{i,j}-U_{(j),i}^{T}V_{j})V_{j}\text{I}(R_{i,j}\neq^{\prime }{⊥}^{\prime }{)+\text{F}}_{j,i,1}+F_{j,i,2}$$

Similarly, we calculate the partial subgradient of *W* with regard to *V_j_* (1 ≤ *j* ≤ *t*):

For 2 ≤ *j* ≤ *t*, define *G_j,_*_1_ = *λsgn*(*V_j_* – *V_j_*_−1_).
For 1 ≤ *j* ≤ *t* – 1, denote *G_j_*_,2_ = – G_*j*+1,1_.
Let *G*_1,1_ = *G_t_*_,2_ = 0, and we have:
  $$\frac{\partial W}{\partial V_{1}}=2\beta V_{1}-2\overset{n}{\underset{i=1}{\text{∑}}}\left(R_{i,1}-U_{\left(1\right),i}^{T}V_{1}\right)U_{\left(1\right),i}\text{I}\left(R_{i,1}\neq^{\prime }{⊥}^{\prime }\right)+G_{1,1}+G_{1,2}$$
  and for *j* > 1:
  $$\frac{\partial W}{\partial V_{j}}=-2\overset{n}{\underset{i=1}{\text{∑}}}\left(R_{i,j}-U_{\left(j\right),i}^{T}V_{j}\right)U_{\left(j\right),i}\text{I}\left(R_{i,j}\neq^{\prime }{⊥}^{\prime }\right)+G_{j,1}+G_{j,2}$$

Finally, with the results above, we present the solution algorithm in Algorithm 1.

## 6. Applications on Missing Sensor Data Imputation

In this section, we evaluate our approach through two large-sized datasets and compare the results with two state-of-the-art algorithms in terms of parametric sensitivity, convergence and missing data recovery performance. The following paragraphs describe the set-up, evaluation methodology and the results obtained. To simplify the parameter tuning, we set *α* = *β* and λ = *γ* in the algorithm implementation.

### 6.1. Evaluation Methodology

Three state-of-the-art algorithms are selected for comparison to the proposed MMP model. The first one is the *k*-nearest neighbor-based imputation model [1]. As a *local model*, for every missing entry, *R_i,j_*, the *knn*method takes the estimation, *R̂_i,j_*, as the mean of the *k* nearest neighbors to it. Let 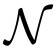
(*x*) be the set consisting of the *k* non-empty entries to *x*; then:

(10) $${\hat{R}}_{i,j}=\frac{1}{k}\sum_{R_{i,l}\in N\left(R_{i,j}\right)}R_{i,l}$$

The second algorithm is the probabilistic principle components analysis model (PPCA) [30,31], which has achieved state-of-the-art performance in the missing traffic flow data imputation problem [31]. Denote the observations of the incomplete matrix, *R*, as *R_o_*. Let *x* ∼ *N*(0*, I*); to estimate the missing values, PPCA first fits the parameters *μ* and *C* with:

(11) $$\left\{\mu *,C*\right\}=\underset{\mu ,C}{\text{arg}}\max\text{Pr}\left(x|R_{o}\right)\sim N\left(x\sigma^{-2}{\left(I+\sigma^{-2}CC^{T}\right)}^{-1}CR_{o},{\left(I+\sigma^{-2}CC^{T}\right)}^{-1}\right)$$

where *σ* is the tunable parameter. Then, with *R_o_*, μ* and C*, it takes the estimation of the missing values (denoted as *R_m_*) as:

(12) $${\hat{R}}_{m}=\underset{R_{m}}{\text{arg}}\max N\;\left(R_{m}|C^{T}x,\sigma^{2}I\right)$$

The third algorithm is the probabilistic matrix factorization model (PMF) [7], one of the most popular algorithms targeting the Netflix matrix completion problem. PMF first seeks the low rank matrices, *U* and *V*, that:

(13) $$\left\{U*,V*\right\}=\underset{U,V}{\text{arg}}\min\overset{n}{\underset{i=1}{\text{∑}}}\overset{t}{\underset{j=1}{\text{∑}}}{\left(R_{i,j}-U_{i}^{T}V_{j}\right)}^{2}\text{I}\left(R_{i,j}\neq^{\prime }{⊥}^{\prime }\right)+\alpha \overset{n}{\underset{i=1}{\text{∑}}}{\left\|U_{i}\right\|}_{2}^{2}+\beta \overset{t}{\underset{j=1}{\text{∑}}}{\left\|V_{j}\right\|}_{2}^{2}$$

then for the missing entry *R_i,j_*, it takes the estimation as

(14) $${\hat{R}}_{i,j}=U_{i}^{T}V_{j}$$

**Algorithm 1:** The Multi-Matrices Factorization Algorithm.
**Input:** matrix *R*; number of the latent features, *d*; learning rates, *η*_1_, *η*_2_, *η*_3_ and *η*_4_; regularization parameters, *α* and λ; threshold *ϵ*.Output: the estimated matrix, *R̂*.// Initialize *U* and *V*.**1**Draw random vectors, *U*_(1),1_, *U*_(1),2_, …, *U*_(1)_*_,n_*, *V*_1_ ∼ *N*(**0**, *I*);**2****for**
*j* = 2; *j* ≤ *t*; *j* + + **do****3** Let *V_j_* = *V_j_*_−1_ + *Z*; here *Z* ∼ Laplace(**0**, 1);**4****end****5****for**
*j* = 2; *j* ≤ *t*; *j* + + **do****6** **for**
*i* = 1; *i* ≤ *n*; *i* + + **do****7**  Let *U*_(_*_j_*_),_*_i_* = *U*_(_*_j_*_-1)_*_,i_* + *Z*; here *Z* ∼ Laplace(**0**, 1);**8** **end****9****end**// Coordinate descent.**10**
$W_{1}=\sum_{i=1}^{n}\sum_{j=1}^{t}{\left(R_{i,j}-U_{(j),i}^{T}V_{j}\right)}^{2}\text{I}(R_{i,j}\neq^{\prime }{⊥}^{\prime })+\alpha \sum_{j=1}^{t}{\left\|U_{(j),1}\right\|}_{2}^{2}+\beta {\left\|V_{1}\right\|}_{2}^{2}+\gamma \sum_{i=1}^{n}\sum_{j=2}^{t}{\left\|U_{(j),i}-U_{(j-1),i}\right\|}_{1}+\lambda \sum_{j=2}^{t}{\left\|V_{j}-V_{j-1}\right\|}_{1}$;**11****12***W*_2_ = inf;**13****while** |*W*_2_ – *W*_1_| > *ϵ*
**do****14** *W*_2_ = *W*_1_;**15** **for**
*i*= 1, 2 …, *n*
**do****16**  Let 
$U_{(1),i}^{\text{new}}=U_{(1),i}-\eta_{1}\frac{\partial W_{1}}{\partial U_{(1),i}}$;**17** **end****18** **for**
*j* > 1 *and i* = 1, 2 …, *n*
**do****19**  Let 
$U_{(j),i}^{\text{new}}=U_{(j),i}-\eta_{2}\frac{\partial W_{1}}{\partial U_{(j),i}}$**20** **end****21** 
$V_{1}^{\text{new}}=V_{1}-\eta_{3}\frac{\partial W_{1}}{\partial V_{1}}$;**22** **for**
*j* = 2 …, *t*
**do****23**  Let 
$V_{j}^{\text{new}}=V_{j}-\eta_{4}\frac{\partial W_{1}}{\partial V_{j}}$;**24** **end****25** Replace all *U*_*(j),i*_*s* with 
$U_{(j),i}^{\text{new}}s$ and *V_j_s* with 
$V_{j}^{\text{new}}s$, recompute *W*_1_;**26****end****27****return**
*R̂*, where 
${\hat{R}}_{j}=U_{\left(j\right)}^{T}V_{j}$;


These three algorithms, as well as the proposed MMF, are employed to perform missing imputations for the incomplete matrix, *R*, on the same datasets.

The testing protocol adopted here is the *Given X* (0 < *X* < 1) protocol [32], *i.e.*, given a matrix, *R*, only *X* percent of its observed entries are revealed, while the remaining observations are concealed to evaluate the trained model. For example, a setting with *X* = 10% means that the algorithm is trained with 10% of the non-missing entries, and the rest of the 90% non-missing ones are held and are to be recovered. In both of the experiments on synthetic and real datasets, the data partition is repeated five times, and the average results, as well as the standard deviations over the five repetitions are recorded.

Similar to many other missing imputation problems [1,3-5,7,13,33-35], we employ the root mean square error (RMSE) to depict the distance between the real values and the estimations: Let *S* = {*s*_1_, *s*_2_, … *s_n_*} be the test dataset and *Ŝ* = {*ŝ*_1_, *ŝ*_2_, … *ŝ_n_*} be the estimated set; here, *ŝ_i_* is the estimation of *s_i_*. Then, the RMSE of the estimation is given by $\sqrt{\frac{1}{n}\sum_{k=1}^{n}{\left(s_{k}-{\hat{s}}_{k}\right)}^{2}}$.

### 6.2. Synthetic Validation

To conduct a synthetic validation of the studied approaches, we randomly draw a 100 × 10, 000 matrix *R* using the procedure detailed in Algorithm 2. The rows in *R* correspond to the areas, *S*_1_, *S*_2_, …, *S_n_*, and the columns correspond to the time. Thus, *R_i,j_* represents the data collected in *S_i_* at time *T_j_*. Notably, the parameter, *r_i_*_,_*_j_*, in Algorithm 2 is used to control the magnitude of the variation happening to *S_i_* from *T_j_*_−1_ to *T_j_*. Combining lines 4 and 5, we have: for *i* ∈ [1, *n*] and *j* ∈ [1, *t*], $\left|\frac{R_{i,j}-R_{i,j-1}}{R_{i,j-1}}\right|=\left|r_{i,j}\right|\leq 0.1$. This constraint ensures that the data collected in *S_i_* does not change too much over time *T_j_*_−1_ to *T_j_*.



**Algorithm 2:** Synthetic Data Generating Procedure.
**1****for**
*i* = 1, 2, …, *n*
**do****2** Draw *R_i_*_,1_ ∼ *N*(0, 1);**3** **for**
*j* = 2, 3, …, *t*
**do****4**  Let *r_i,j_* ∼ Uniform(−0.1, 0.1);**5**  *R_i,j_* ← *R_i,j_*_−1_ + *r_i,j_R_i,j_*_−1_;**6** **end****7****end**


**Figure 2.**
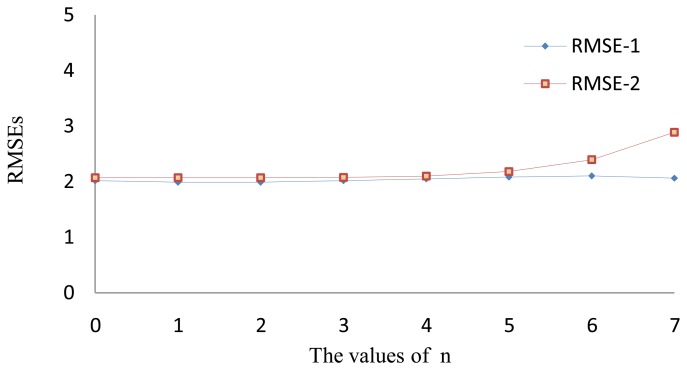
Empirical studies on parameter sensitivity.

We first evaluate the sensitivity of the proposed algorithm to the regularization parameters, *α* and λ. Half of the entries in *R* are randomly selected as testing data and recovered using the remaining 50% as the training data. Namely, we take *X* = 50% in the *Given X* protocol. In the experiments, we first fix *α* = 0.01, tune λ via λ = 0.01 × 2*^n^* (*n* = 0, 1, … 7) and, then, do the reverse by changing *α* via *α* = 0.01 × 2*^n^* (*n* = 0, 1, … 7), but setting λ = 0.01. The average RMSEs with the same parameter settings on different data partitions are summarized in Figure 2.

In Figure 2, the RMSE-1 curve represents the recovery errors obtained by fixing *α* and changing the value of λ. The RMSE-2 curve corresponds to the errors with different *α* values and fixed λ. We can see that even when λ is expanded by more than 100 times (2^7^ = 128), the RMSE still remains stable. A similar result also appears in the experiments on the parameter, *α*, where a significant change of the RMSE only occurs when *n* is greater than six, *i.e.*, when *α* is expanded more than 60 times.

The second experiment we conduct is to study the prediction ability of the proposed algorithm, as well as that of the comparison algorithms. In the *Given X* protocol, we set *X* = 10%, 20%, …, 90% in sequence. Then, for each *X* value, we perform missing imputations via our algorithm and the comparison algorithms. In the all implementations, we set *k* = 5 for the *knn* model. As with the MF-based algorithms, we examine their performance with respect to the latent feature dimension *d* = 10 and *d* = 30 respectively. Furthermore, in the implementation of MMF, we fix *α* = λ = 0.1, *η*_1_ = *η*_2_ = *η*_3_ = *η*_4_ = 0.01. All results are summarized in Table 1.

As shown in Table 1, when X is large, e.g., *X* ≥ 50%, *knn* is competitive with the matrix factorization methods, while in the other situations, the MF methods outperform it significantly. In terms of the MF-based methods, we find that our algorithm outperforms PPCA significantly in all settings. The RMSEs of our algorithm are at most roughly 20% of that of PPCA. Specifically, for *X* ∈ [30%; 80%], the RMSEs of the proposed algorithm are even only 10% of that of PPCA. We can also observe that the parameter, d, has a different impact on the performances of our algorithm and PPCA: When d changes from 10 to 30, most of the RMSEs of PPCA increase evidently, while for our algorithm, the RMSEs are reduced by roughly 5%.

When compared with PMF, our algorithm also performs better in most of the settings: PMF achieves lower RMSEs than MMF only in two cases, in which *d* = 10, *X* = 60% and *d* = 10; *X* = 80% respectively. Another interesting finding is that the promotion of the feature number, *d* (from 10 to 30), has little impact on the performance of PMF.

**Figure 3.**
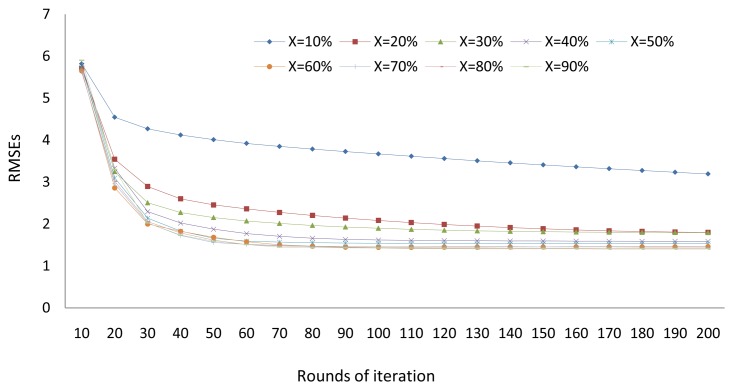
Empirical studies on convergence speed.

We also exam the convergence speed of the proposed algorithm. In the missing recovery experiments conducted above, for each *X* setting, we record the average RMSE of the recovered results after every 10 iterations of all data partitions. We can see from Figure 3 that, for all *X* values, the errors drop dramatically in the first 20 iterations and remain stable after the first 100 iterations. We can conclude that the proposed algorithm converges to the local optimization solutions after around 100 iterations.

### 6.3. Application to Impute the Missing Traffic Speed Values

To evaluate the feasibility of the proposed approach on real-world applications, in this section, we conduct another experiment on a traffic speed dataset, which was collected in the urban road network of Zhuhai City [36], China, from April 1, 2011 to April 30, 2011. The data matrix, *R*, consists of 1,853 rows and 8,729 columns. Each row corresponds to a road, and each column corresponds to a five minute-length time interval. All columns are arranged in ascending order of time. An entry, *R_i,j_* (1 ≤ *i* ≤ 1, 853,1 ≤ *j* ≤ 8729), in *R* is the aggregate mean traffic speed of the *ith* road in the *jth* interval. Since all the data in *R* are collected by floating cars [37], the value of *R_i,j_* could be *missing* if there is no car on the *i* th road in the *j* th time interval. Our statistics show that in *R*, there are nearly half of the entries, *i.e.*, eight million entries are missing values.

We perform missing imputation on matrix *R* using the studied algorithms with parameter settings *k* = 5 and *d* = 10. In the implementation of MMF, we fix *α* = 0.25, λ = 0.5, *η*_1_ = *η*_2_ = *η*_3_ = *η*_4_ = 0.5. We summarize all results in Table 2, from which both the feasibility and effectiveness of MMF are well verified. In detail, when *X* is large enough, e.g., *X* ≥ 80%, *knn* is competitive, while in the other cases, *knn* cannot work as well as the MF based algorithms. As for the MF algorithms, we see that the proposed MMF outperforms PPCA and PMF in all *X* settings. Particularly, when the observations are few (*X* = 10% and *X* = 20%), the errors of our algorithm reduce by 33% compared to those of PPCA and by 10% compared to those of PMF, respectively. When *X* > 20%, the RMSE differences between PPCA and our algorithm tend to be slight, but the overall errors of PPCA are roughly 3% ∼ 5% higher than those of MMF. For PMF, the RMSEs remain about 10% higher than MMF in all settings.

## 7. Conclusion

Missing estimation is one of the main concerns in current studies on sensor data-based applications. In this work, we formulate the estimation problem as a matrix completion one and present a multi-matrices factorization model to address it. In our model, each column, *R_j_*, of the target matrix, *R*, is approximated by the product of a spatial feature matrix, *U*_(_*_j_*_)_, and a temporal feature vector, *V_j_*. Both *U_j_* and *V_j_* are time dependent, and hence, their product accommodates the ability to describe the time variant sensor data. We also present a solution algorithm to the factorization model. Empirical studies on a synthetic dataset and real sensor data show that our approach outperforms the comparison algorithms.

Reviewing the present work, it is notable that the proposed model only incorporates the temporal structure information, while the information on the spatial structure is disregarded, e.g., the data collected in two adjacent areas, *S_k_* and *S_l_*, should be close to each other. Hence, our next step is to extend our model with more complex structured data.

## Figures and Tables

**Table 1.** Recovery errors on the synthetic dataset (mean ± std).

		10%	20%	30%	40%	50%	60%	70%	80%	90%
	knn	13.41 ± 0.62	6.80 ± 0.19	4.44 ± 0.06	3.12 ± 0.05	2.27 ± 0.02	1.81 ± 0.09	1.72 ± 0.01	1.69 ± 0.05	1.62 ± 0.06

d = 10	PPCA	17.09 ± 2.08	20.24 ± 1.30	22.75 ± 1.32	23.96 ± 2.19	20.79 ± 1.00	16.67 ± 1.34	18.68 ± 0.81	11.98 ± 0.70	5.11 ± 3.10
	PMF	3.23 ± 0.23	3.33 ± 0.19	3.29 ± 0.12	3.34 ± 0.07	3.29 ± 0.09	1.69 ± 0.04	1.83 ± 0.02	1.81 ± 0.03	1.85 ± 0.04
	MMF	3.07 ± 0.07	2.21 ± 0.10	2.14 ± 0.09	1.98 ± 0.06	1.93 ± 0.06	1.92 ± 0.04	1.75 ± 0.03	1.84 ± 0.02	1.80 ± 0.03

d = 30	PPCA	19.65±3.01	22.48 ± 0.86	24.86 ± 1.54	23.99 ± 0.61	22.67 ± 0.49	20.22 ± 1.46	17.53 ± 0.70	14.23 ± 1.70	11.14 ± 1.72
	PMF	3.20 ± 0.21	3.32 ± 0.13	3.35 ± 0.09	3.36 ± 0.11	3.31 ± 0.07	1.78 ± 0.04	1.81 ± 0.02	1.79 ± 0.02	1.86 ± 0.11
	MMF	3.06 ± 0.05	2.17 ± 0.08	2.07 ± 0.05	1.94 ± 0.03	1.74 ± 0.03	1.62 ± 0.02	1.64 ± 0.03	1.65 ± 0.01	1.69 ± 0.03

**Table 2.** Recovery errors on the transportation dataset (mean ± std).

	10%	20%	30%	40%	50%	60%	70%	80%	90%
knn	40.47 ± 0.02	31.79 ± 0.01	25.41 ± 0.02	21.35 ± 0.00	18.33 ± 0.00	15.89 ± 0.01	13.73 ± 0.01	11.67 ± 0.00	9.45 ± 0.02
PPCA	17.90 ± 0.01	17.36 ± 0.01	13.00 ± 0.02	12.25 ± 0.01	11.47 ± 0.01	11.31 ± 0.03	11.19 ± 0.02	11.14 ± 0.04	11.16 ± 0.10
PMF	14.41 ± 0.01	12.83 ± 0.03	12.43 ± 0.01	12.33 ± 0.02	12.36 ± 0.01	12.35 ± 0.01	12.13 ± 0.00	11.99 ± 0.03	11.96 ± 0.02
MMF	11.79 ± 0.02	11.51 ± 0.01	11.43 ± 0.01	11.05 ± 0.02	11.05 ± 0.01	11.01 ± 0.00	10.83 ± 0.01	10.69 ± 0.01	10.70 ± 0.02
